# Oral Small-Molecule Therapies Versus Biologic Agents in Moderate-to-Severe Ulcerative Colitis: A Systematic Review of Pivotal Phase 3 Trials

**DOI:** 10.7759/cureus.109528

**Published:** 2026-05-23

**Authors:** Okasha Zulfiqar, Talha Ali, Ghulam Mohayy Ud Din, Zaheer Ud Din Babar, Shabir Zarif

**Affiliations:** 1 Internal Medicine, Mayo Hospital, Lahore, PAK; 2 Cardiac Surgery, Mayo Hospital, Lahore, PAK; 3 Internal Medicine, Indus Medical College, Tando Muhammad Khan, PAK; 4 Internal Medicine, Nishtar Medical University, Multan, PAK

**Keywords:** biologics, induction and maintenance therapy, randomized controlled trials, small-molecule therapies, sphingosine-1-phosphate receptor modulators, ulcerative colitis

## Abstract

This systematic review evaluates the comparative efficacy and safety of oral small-molecule therapies and biologic agents in adults with moderate-to-severe ulcerative colitis (UC). Evidence from eight landmark publications encompassing multiple pivotal phase 3 randomized controlled trials was synthesized to assess induction and maintenance outcomes across distinct therapeutic mechanisms, including intracellular cytokine signaling inhibition, immune trafficking modulation, and extracellular pathway blockade. The findings suggest that oral small-molecule therapies demonstrate clinically meaningful efficacy comparable to established biologic agents while offering practical advantages related to oral administration and mechanistic diversity. Distinct differences in induction and maintenance performance across therapeutic classes highlight the importance of goal-directed therapy selection, individualized risk stratification, and mechanism-based treatment sequencing rather than class cycling alone. Overall, this review underscores the evolving role of oral therapies as integral components of contemporary UC management while emphasizing the need to balance efficacy, safety considerations, and patient-centered treatment priorities.

## Introduction and background

Ulcerative colitis (UC) is a chronic, relapsing inflammatory bowel disease characterized by continuous mucosal inflammation of the colon and recurrent episodes of symptomatic intestinal inflammation [1,2]. The disease follows a variable course marked by periods of remission and exacerbation, significantly impairing quality of life and imposing a substantial burden on healthcare systems worldwide. Despite advances in medical therapy, a considerable proportion of patients with moderate-to-severe UC experience inadequate disease control, loss of response, or treatment-limiting adverse effects, highlighting the need for more effective and durable therapeutic strategies [3].

Historically, the management of moderate-to-severe UC has relied on biologic agents targeting key inflammatory pathways, most notably tumor necrosis factor-alpha (TNF-α), integrin-mediated leukocyte trafficking, and IL signaling. Anti-TNF agents such as infliximab and adalimumab revolutionized UC treatment by demonstrating the ability to induce and maintain remission, reduce hospitalization rates, and delay colectomy [4,5]. Subsequent development of gut-selective agents like vedolizumab and cytokine-directed therapies such as ustekinumab further expanded the therapeutic armamentarium, offering improved safety profiles and alternative mechanisms of action for patients with prior biologic failure. Nevertheless, biologic therapies are limited by several challenges, including parenteral administration, immunogenicity, secondary loss of response, delayed onset of action, and high treatment costs [6].

In recent years, novel oral small-molecule therapies have emerged as an important addition to UC management, offering targeted immunomodulation with distinct pharmacologic advantages. Janus kinase (JAK) inhibitors are oral agents that block intracellular inflammatory signaling pathways involved in immune activation, whereas sphingosine-1-phosphate (S1P) receptor modulators reduce inflammatory cell trafficking to the gastrointestinal tract by limiting lymphocyte migration from lymphoid tissues. JAK inhibitors, including tofacitinib and upadacitinib, act by interrupting intracellular cytokine signaling pathways involved in lymphocyte activation and inflammatory amplification [7,8]. These agents provide rapid onset of action and oral administration, features that are particularly appealing in clinical practice. Similarly, S1P receptor modulators such as ozanimod and etrasimod reduce intestinal inflammation by selectively inhibiting lymphocyte egress from lymphoid tissues, thereby limiting immune cell trafficking to the gut [9]. Together, these oral therapies represent a shift from extracellular cytokine blockade toward intracellular signaling and immune trafficking modulation.

While both biologic therapies and oral small-molecule agents have demonstrated efficacy in randomized controlled trials, direct head-to-head comparisons between these therapeutic classes remain limited [10]. In clinical trials, induction therapy generally refers to the initial short-term treatment phase aimed at achieving disease remission, whereas maintenance therapy refers to longer-term treatment intended to sustain remission and prevent relapse. As a result, clinicians are often required to make treatment decisions based on indirect comparisons across trials with differing study designs, patient populations, induction and maintenance frameworks, and outcome definitions. Furthermore, the expanding number of available therapies has increased the complexity of treatment sequencing, particularly in patients with prior biologic exposure or high-risk disease features. The objective of this systematic review is to compare the efficacy and safety of oral small-molecule therapies, including JAK inhibitors and S1P receptor modulators, with established biologic agents such as anti-tumor necrosis factor therapies, anti-integrin agents, and IL-12/23 inhibitors in adults with moderate-to-severe UC. By synthesizing evidence from pivotal phase 3 randomized controlled trials, this review aims to evaluate clinical and endoscopic outcomes during both induction and maintenance therapy, thereby providing a focused and clinically relevant framework to guide therapeutic selection and mechanism-based treatment sequencing in contemporary UC management. This review, therefore, synthesizes phase 3 trial data indirectly, comparing remission and safety outcomes across therapeutic drug classes.

## Review

Materials and methods

Study Design and Reporting Standards

This systematic review was conducted in accordance with the PRISMA 2020 guidelines [11]. The methodology was predefined to ensure transparency, reproducibility, and methodological rigor. Although the review protocol was not prospectively registered in the PROSPERO database, predefined eligibility criteria and methodological procedures were established prior to study selection and data extraction. Given the absence of sufficient head-to-head trials directly comparing oral small-molecule therapies with biologic agents in UC, a qualitative synthesis of pivotal randomized controlled trials was undertaken.

Research Question and Population, Intervention, Comparator, and Outcomes (PICO) Framework

The research question was formulated using the PICO framework [12]. The population comprised adults with moderate-to-severe UC. The interventions included oral small-molecule therapies, specifically JAK inhibitors and S1P receptor modulators. The comparators were established biologic agents, including anti-tumor necrosis factor therapies, anti-integrin agents, and IL-12/23 inhibitors. The primary outcomes of interest were clinical remission and endoscopic improvement during induction and maintenance therapy, while secondary outcomes included clinical response and safety-related outcomes.

Eligibility Criteria

Randomized controlled trials were eligible for inclusion if they evaluated adults aged 18 years or older with moderate-to-severe UC and assessed the efficacy of either oral small-molecule therapies or biologic agents as induction and/or maintenance treatment. Only phase 3 randomized, double-blind, placebo-controlled trials were included to ensure a high level of internal validity. For the purpose of this review, “pivotal” trials were defined as major phase 3 studies that served as the primary basis for regulatory approval and incorporation into contemporary clinical practice guidelines. Studies were required to report standardized clinical outcomes based on the Mayo score or adapted Mayo score [13]. Trials involving pediatric populations, observational studies, post hoc analyses, real-world registry data, non-randomized designs, or studies focusing exclusively on Crohn’s disease were excluded. Only articles published in the English language were considered.

Information Sources and Search Strategy

A structured literature search was performed using PubMed/MEDLINE, Google Scholar, and the Cochrane Library and was supplemented by manual screening of references from relevant articles. The literature search included studies published from database inception through April 2026, and the final search was executed on April 15, 2026. The search strategy focused on identifying pivotal phase 3 randomized controlled trials evaluating oral small-molecule therapies and biologic agents in moderate-to-severe UC. Database-specific search strategies were developed using combinations of keywords, Medical Subject Headings (MeSH), and Boolean operators (AND/OR). Priority was given to trials that served as the basis for regulatory approval and contemporary guideline recommendations. A summary of the search strategy across electronic databases is presented in Table 1.

**Table 1:** Summary of the literature search strategy across electronic databases

Database	Search terms/MeSH terms	Boolean operators/strategy
PubMed/MEDLINE	“Ulcerative Colitis” [MeSH], ulcerative colitis, tofacitinib, upadacitinib, ozanimod, etrasimod, infliximab, adalimumab, vedolizumab, ustekinumab, induction, maintenance, randomized controlled trial	AND, OR
Cochrane Library	ulcerative colitis, biologic therapy, small-molecule therapy, JAK inhibitor, S1P receptor modulator, randomized trial	AND, OR
Google Scholar	ulcerative colitis AND biologic agents AND oral small molecules AND phase 3 trial	AND

Study Selection

Two reviewers independently screened titles and abstracts to assess eligibility. Full-text articles were subsequently reviewed to confirm inclusion based on the predefined criteria. Any discrepancies during study selection were resolved through discussion and consensus. The final selection comprised eight landmark publications encompassing multiple pivotal phase 3 randomized controlled trials representing both oral small-molecule therapies and biologic agents in moderate-to-severe UC.

Data Extraction

Data extraction was performed using a standardized form designed prior to study selection. Extracted data included study author and year of publication, intervention class, study design and phase, sample size, prior therapy exposure, induction and maintenance duration, primary efficacy outcomes, and key safety findings. Data extraction focused on outcomes reported during both induction and maintenance phases to allow consistent comparison across therapeutic classes.

Risk of Bias Assessment

The Cochrane Risk of Bias 2 (RoB 2) tool [14] was used to assess the methodological quality of the included randomized controlled trials. Each study was evaluated across five domains: randomization process, deviations from intended interventions, missing outcome data, measurement of outcomes, and selection of reported results. Each domain was classified as low risk, some concerns, or high risk of bias, leading to an overall risk-of-bias judgment for each study. Disagreements in bias assessment were resolved by discussion. Overall, the included trials demonstrated a low risk of bias, with some concerns primarily related to attrition during long-term maintenance phases.

Data Synthesis and Analysis

Given the substantial clinical and methodological heterogeneity among the included trials, including differences in induction and maintenance designs, treatment sequencing, prior biologic exposure, endpoint definitions, follow-up duration, and responder re-randomization strategies, a quantitative synthesis was not considered methodologically appropriate. Accordingly, a qualitative narrative synthesis was performed to descriptively compare efficacy and safety outcomes across therapeutic classes. Although network meta-analysis and indirect treatment comparison methodologies are commonly used in the absence of head-to-head trials, these approaches were not undertaken because of significant cross-trial heterogeneity and variability in study populations, outcome measures, and trial structures, which could limit the validity and interpretability of pooled indirect comparisons. Therefore, the review was designed to provide a focused narrative synthesis of pivotal phase 3 trial evidence rather than pooled quantitative comparative estimates.

Ethical Considerations

As this study was a systematic review of previously published data, ethical approval and informed consent were not required.

Results

Study Selection Process

**Figure 1:**
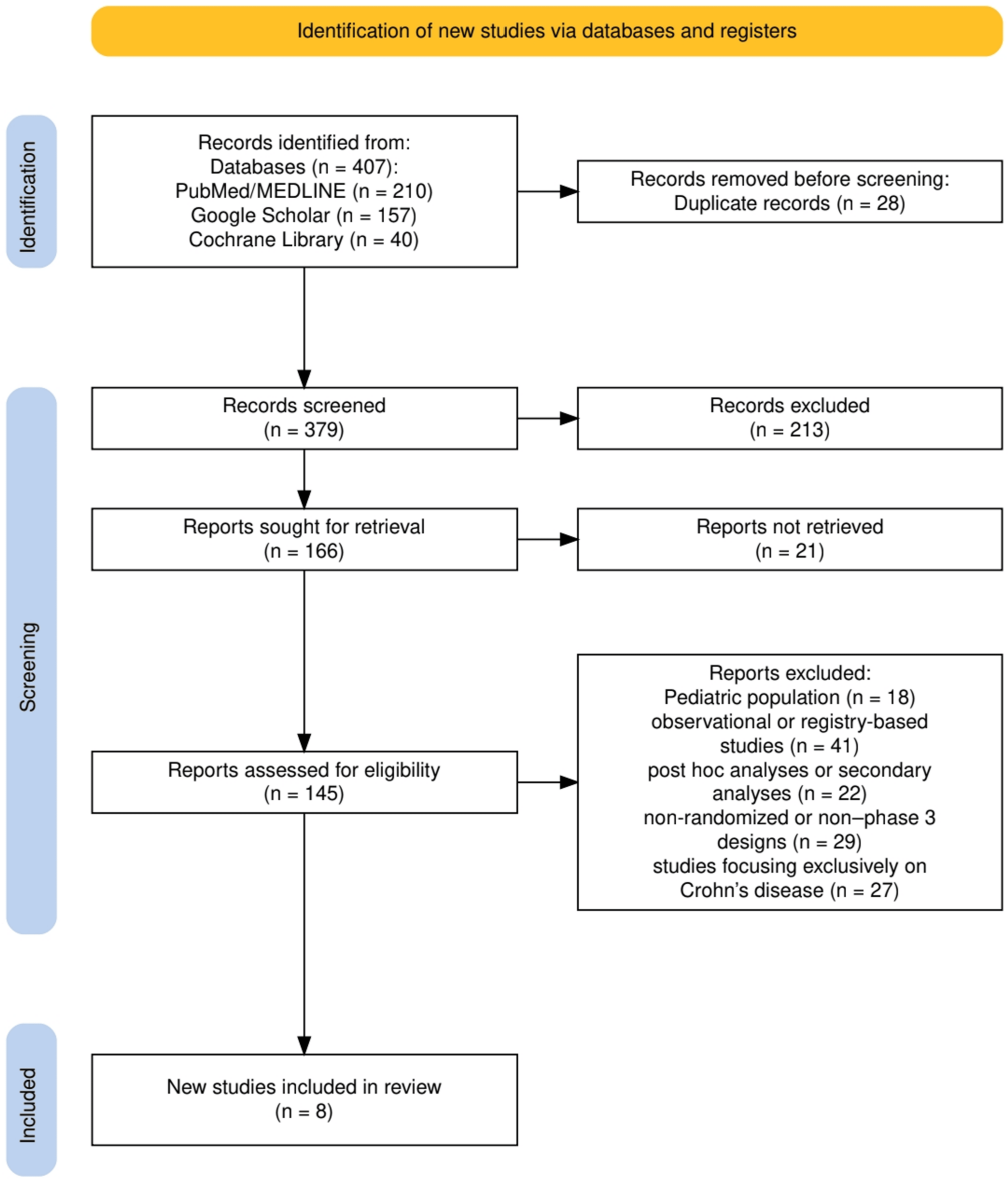
PRISMA 2020 flow diagram illustrating the study selection process for the inclusion of pivotal phase 3 randomized controlled trials comparing oral small-molecule therapies and biologic agents in moderate-to-severe UC UC, ulcerative colitis

The study selection process is illustrated in Figure 1. A total of 407 records were initially identified through database searching, including PubMed/MEDLINE, Google Scholar, and the Cochrane Library. After removal of 28 duplicate records, 379 studies were screened based on titles and abstracts. Of these, 213 records were excluded for not meeting the inclusion criteria, and 166 full-text reports were sought for retrieval. Following this step, 21 reports could not be retrieved, leaving 145 articles assessed for full-text eligibility. Among these, 137 studies were excluded for predefined reasons, including pediatric populations, observational or registry-based designs, post hoc or secondary analyses, non-randomized or non-phase 3 studies, and studies focusing exclusively on Crohn’s disease. Ultimately, eight landmark publications encompassing multiple pivotal phase 3 randomized controlled trials met all eligibility criteria and were included in the final qualitative narrative synthesis, as depicted in the PRISMA flow diagram in Figure 1.

Characteristics of the Selected Studies

The characteristics of the included studies are summarized in Table 2. A total of eight landmark publications encompassing multiple pivotal phase 3 randomized, double-blind, placebo-controlled trials were included, enrolling adults with moderate-to-severe UC who had an inadequate response to conventional therapy, with several studies also including biologic-experienced populations. The evidence base comprised both oral small-molecule therapies, including JAK inhibitors and S1P receptor modulators, and established biologic agents targeting tumor necrosis factor, integrin-mediated lymphocyte trafficking, and IL-12/23 pathways. Induction periods ranged from six to 12 weeks, while maintenance phases extended up to 52 weeks, allowing assessment of both short-term efficacy and long-term disease control. Across studies, clinical remission and response were consistently defined using Mayo score-based criteria, facilitating qualitative comparison despite differences in trial design and therapeutic class. Overall, the included trials were large, multicenter, and globally representative, providing a robust and methodologically rigorous foundation for narrative interpretation of efficacy and safety outcomes.

**Table 2:** Characteristics of pivotal phase 3 randomized controlled trials evaluating oral small-molecule therapies and biologic agents in adults with moderate-to-severe UC ^*^ Maintenance cohort sizes represent induction responders who were subsequently re-randomized into maintenance phases according to the original trial protocols; therefore, differences between induction and maintenance population sizes should not be interpreted solely as study attrition or dropout. ACT, active ulcerative colitis trials; AEs, adverse events; anti-TNF, anti-tumor necrosis factor; CPK, creatine phosphokinase; JAK, Janus kinase; JAK1, Janus kinase 1; S1P, sphingosine-1-phosphate; TNF, tumor necrosis factor; UC, ulcerative colitis

Study (author and year)	Landmark trial acronym	Intervention (class)	Study design/phase	Population (n)	Prior therapy	Induction duration	Maintenance duration	Primary efficacy outcome(s)	Key safety findings
Sandborn et al. (2017) [15]	OCTAVE Induction 1 and 2/OCTAVE Sustain	Tofacitinib (JAK inhibitor)	Phase 3, randomized, double-blind, placebo-controlled trials	Adults with moderate-to-severe UC (induction 1: n = 598; induction 2: n = 541; maintenance: n = 593)	Prior conventional therapy or anti-TNF failure	8 weeks	52 weeks	Clinical remission at 8 weeks (induction) and 52 weeks (maintenance)	Higher rates of overall and serious infections during induction; increased herpes zoster and lipid levels; rare nonmelanoma skin cancer and cardiovascular events
Danese et al. (2022) [16]	U-ACHIEVE/U-ACCOMPLISH	Upadacitinib (JAK1 inhibitor)	Phase 3, multicenter, randomized, double-blind, placebo-controlled^*^	Adults with moderate-to-severe UC (induction: n = 996; maintenance: n = 451)	Conventional and biologic-experienced patients	8 weeks	52 weeks	Clinical remission at weeks 8 and 52	Acne, CPK elevation, herpes zoster; low rates of serious infections and thromboembolism
Sandborn et al. (2021) [17]	TRUE NORTH	Ozanimod (S1P receptor modulator)	Phase 3, multicenter, randomized, double-blind, placebo-controlled	Adults with moderate-to-severe UC (induction: n = 1012; maintenance: n = 457)	Conventional and biologic-experienced patients	10 weeks	52 weeks	Clinical remission during induction and maintenance	Similar infection rates during induction; slightly higher infections during maintenance; elevated liver aminotransferases; low serious infection rates
Sandborn et al. (2023) [18]	ELEVATE UC 12/ELEVATE UC 52	Etrasimod (S1P receptor modulator)	Two phase 3, multicenter, randomized, double-blind, placebo-controlled trials	Adults with moderate-to-severe UC (ELEVATE UC 52: n = 433; ELEVATE UC 12: n = 354)	Prior approved UC therapy failure or intolerance	12 weeks	40 weeks (total 52 weeks in UC 52)	Clinical remission at weeks 12 and 52	Higher overall AEs versus placebo; no deaths or malignancies; well tolerated
Rutgeerts et al. (2005) [19]	ACT 1/ACT 2	Infliximab (anti-TNF biologic)	Two phase 3, randomized, double-blind, placebo-controlled trials	Adults with moderate-to-severe UC (ACT 1 and 2: n = 728 total)	Inadequate response to conventional therapy	8 weeks	Up to 54 weeks (ACT 1)/30 weeks (ACT 2)	Clinical response based on Mayo score at weeks 8, 30, and 54	Acceptable safety profile; infusion-related reactions and infections reported
Sandborn et al. (2012) [20]	ULTRA 2	Adalimumab (anti-TNF biologic)	Phase 3, randomized, double-blind, placebo-controlled trial	Adults with moderate-to-severe UC (n = 494)	Conventional therapy failure; stratified by prior anti-TNF exposure	8 weeks	52 weeks	Clinical remission at weeks 8 and 52	Serious AEs ~12%; serious infections ≤2%; rare malignancies reported
Feagan et al. (2013) [21]	GEMINI 1	Vedolizumab (anti-integrin biologic)	Two integrated phase 3, randomized, double-blind, placebo-controlled trials	Adults with active moderate-to-severe UC (induction: n = 895; maintenance responders re-randomized)	Conventional and biologic-experienced patients	6 weeks	Up to 52 weeks	Clinical response at week 6; clinical remission at week 52	AE rates similar to placebo; favorable safety profile
Sands et al. (2019) [22]	UNIFI	Ustekinumab (IL-12/23 inhibitor biologic)	Phase 3, randomized, double-blind, placebo-controlled trials	Adults with moderate-to-severe UC (induction: n = 961; maintenance responders re-randomized)	Conventional and biologic-experienced patients	8 weeks	44 weeks (total exposure up to 52 weeks)	Clinical remission at weeks 8 (induction) and 44 (maintenance)	Serious AEs similar to placebo; low rates of malignancy and mortality

Risk of Bias Assessment

The risk of bias assessment for the included studies is presented in Table 3. Overall, the pivotal phase 3 randomized controlled trials demonstrated a low risk of bias, reflecting robust methodological quality across key domains, including randomization procedures, blinding, and outcome measurement. Some studies were judged to have minor concerns, primarily related to missing outcome data during long-term maintenance phases and the use of re-randomized responder designs, which may introduce attrition-related bias. However, outcome definitions were prespecified and consistently reported, and there was no evidence of selective outcome reporting across trials. Importantly, no study was assessed as having a high risk of bias in any domain, supporting the internal validity of the evidence base and the reliability of the synthesized conclusions.

**Table 3:** Risk of bias assessment of included pivotal phase 3 randomized controlled trials using the Cochrane RoB 2 tool RoB 2, Risk of Bias 2

Study (author and year)	Randomization process	Deviations from intended interventions	Missing outcome data	Measurement of outcome	Selection of reported results	Overall RoB 2
Sandborn et al. (2017) [15]	Low	Low	Low	Low	Low	Low
Danese et al. (2022) [16]	Low	Low	Low	Low	Low	Low
Sandborn et al. (2021) [17]	Low	Low	Some concerns	Low	Low	Some concerns
Sandborn et al. (2023) [18]	Low	Low	Some concerns	Low	Low	Some concerns
Rutgeerts et al. (2005) [19]	Low	Low	Some concerns	Low	Low	Some concerns
Sandborn et al. (2012) [20]	Low	Low	Some concerns	Low	Low	Some concerns
Feagan et al. (2013) [21]	Low	Low	Some concerns	Low	Low	Some concerns
Sands et al. (2019) [22]	Low	Low	Some concerns	Low	Low	Some concerns

Discussion

Oral Small Molecules in Contemporary UC Therapy

This systematic review adds to the existing literature by reframing oral small-molecule therapies as true therapeutic alternatives rather than adjuncts to biologics in moderate-to-severe UC. Across pivotal phase 3 trials, oral agents demonstrated clinically meaningful induction and maintenance efficacy that is comparable in magnitude to established biologics. For instance, JAK inhibitors achieved induction remission rates of approximately 17-34% at eight weeks and maintenance remission rates reaching 40-52% at 52 weeks, while S1P receptor modulators achieved induction remission rates of 18-27% and maintenance remission rates of 32-37%. These outcomes are within the same efficacy range as anti-TNF and non-TNF biologics, which reported induction remission or response rates of 16-47% and maintenance remission rates of 38-45% at one year. Importantly, oral therapies achieved these outcomes without the need for parenteral administration or concerns related to immunogenicity. However, these benefits should be interpreted alongside the distinct adverse event profiles associated with oral agents, including herpes zoster infections, lipid elevations, hepatic enzyme abnormalities, and thromboembolic concerns reported with certain therapies. Collectively, these findings support a paradigm shift in which treatment selection may increasingly be guided by patient preference, speed of disease control, mechanistic suitability, and individualized risk stratification rather than a biologic-first hierarchy.

Mechanistic Drivers of Therapeutic Response

A key novel contribution of this review is the mechanism-based interpretation of efficacy patterns observed across therapeutic classes. JAK inhibitors exert broad intracellular cytokine signaling inhibition, which likely explains their rapid onset of action, reflected by higher early induction remission rates within eight weeks [23]. In contrast, S1P receptor modulators act by selectively limiting lymphocyte egress from lymphoid tissue, resulting in a more gradual but sustained immunomodulatory effect, consistent with their balanced induction and maintenance remission rates over 52 weeks. Biologic agents, including anti-TNF, anti-integrin, and IL-12/23 inhibitors, function through extracellular pathway blockade and demonstrated strong maintenance durability, with remission rates of approximately 38-45% at 44-52 weeks, albeit with comparatively slower induction responses [19,21,22]. These mechanistic differences suggest that therapeutic outcomes are not solely drug-specific but pathway-dependent, underscoring the importance of aligning treatment choice with clinical priorities such as rapid symptom control, long-term disease stability, and patient-specific risk profiles. By emphasizing mechanism-driven efficacy rather than numerical comparisons alone, this review provides a clinically relevant framework to support personalized treatment strategies in UC.

Induction-Maintenance Trade-Offs

An important clinical insight emerging from this review is that therapeutic performance differs meaningfully between induction and maintenance phases, suggesting that treatment choice should be context-dependent rather than uniform. JAK inhibitors generally demonstrated more rapid induction of remission, likely reflecting their broad intracellular cytokine signaling inhibition and fast pharmacodynamic effects [24]. In contrast, S1P receptor modulators exhibited a more balanced efficacy profile across induction and maintenance phases, consistent with gradual immune trafficking modulation. Biologic agents, particularly anti-integrin and IL-12/23 inhibitors, demonstrated comparatively slower induction responses but durable long-term maintenance efficacy [9]. These phase-specific therapeutic patterns suggest that oral JAK inhibitors may be preferentially suited for patients requiring rapid disease control, whereas S1P modulators and biologics may be better positioned for sustained long-term disease stability. Collectively, these findings highlight the importance of goal-directed and mechanism-informed therapy selection in UC.

Oral Versus Injectable Therapies

The emergence of effective oral small-molecule therapies represents a paradigm shift in UC management, extending beyond efficacy to patient-centered considerations. Oral agents offer several practical advantages, including ease of administration, avoidance of infusion-related logistics, reduced risk of immunogenicity, and predictable pharmacokinetics that are independent of body weight or antibody formation [25]. In contrast, biologic therapies require intravenous or subcutaneous administration, are susceptible to antidrug antibody development, and may be associated with infusion reactions or injection-site adverse events, potentially compromising long-term treatment adherence. Importantly, the comparable induction and maintenance efficacy observed between oral agents and biologics in pivotal trials challenges the traditional perception of oral therapies as salvage options. Instead, these findings support the repositioning of oral small molecules as legitimate first-line or early-line advanced therapies, particularly for patients who prioritize convenience, rapid symptom relief, or avoidance of parenteral treatment.

Safety Profiles and Risk Stratification

A nuanced interpretation of safety data across therapeutic classes underscores the importance of distinguishing class-specific effects from drug-specific risks. JAK inhibitors were associated with higher rates of herpes zoster infection and lipid elevations, reflecting systemic cytokine pathway inhibition, while S1P receptor modulators were more commonly linked to elevated liver aminotransferases and, in earlier studies, transient heart rate effects related to lymphocyte trafficking modulation [9]. Importantly, JAK inhibitors also carry FDA boxed warnings regarding major adverse cardiovascular events, malignancy, thrombosis, and serious infections, which have become central considerations in therapeutic decision-making and patient selection. Biologic therapies demonstrated safety profiles characterized by infection risk and immunogenicity, including infusion reactions and antidrug antibody formation, particularly with anti-TNF agents. Despite these differences, serious adverse events were generally infrequent across pivotal phase 3 trials, and overall safety profiles remained acceptable within appropriately selected patient populations. These findings emphasize that treatment decisions should move beyond binary notions of “safe” versus “unsafe” and instead adopt a risk-matched approach, aligning therapy choice with individual patient comorbidities, infection risk, cardiovascular profile, malignancy risk, and long-term treatment goals.

Therapeutic Positioning and Treatment Sequencing

The findings of this review support a more flexible positioning of oral small-molecule therapies within contemporary UC treatment algorithms. Following failure of conventional therapies, oral agents may serve as effective alternatives to initiating biologic therapy, particularly in patients prioritizing rapid symptom control or oral administration. Additionally, the comparable efficacy of oral small molecules and biologics suggests that these agents may be considered as first-line advanced therapies rather than being reserved exclusively for biologic-refractory disease [26]. In patients with prior biologic exposure, especially those experiencing secondary loss of response, switching to a therapy with a distinct mechanism of action, such as transitioning from extracellular cytokine blockade to intracellular signaling inhibition or immune trafficking modulation, may offer greater clinical benefit than cycling within the same biologic class. This mechanism-based sequencing approach provides a rational, evidence-informed framework for optimizing treatment outcomes and minimizing therapeutic redundancy.

Strengths and Limitations

This review has several strengths that enhance its clinical and methodological relevance. It focuses exclusively on pivotal phase 3 randomized controlled trials, ensuring high internal validity and minimizing bias associated with lower-quality evidence. Outcome measures were relatively homogeneous across studies, predominantly relying on standardized Mayo score-based definitions, which facilitated meaningful cross-trial comparison. Furthermore, the inclusion of trials published in high-impact journals such as The New England Journal of Medicine and The Lancet underscores the rigor of the evidence base and reflects therapies that have shaped clinical practice. Nonetheless, certain limitations merit consideration. Direct head-to-head comparisons between oral small molecules and biologics are lacking; however, this reflects the current state of the literature rather than a methodological shortcoming of the review. The absence of real-world data limits generalizability, although the focus on controlled trial settings was intentional to prioritize efficacy and safety assessment. Finally, variability in induction duration across studies may introduce heterogeneity, but all included trials evaluated clinically relevant induction and maintenance endpoints, supporting the validity of comparative interpretation.

Clinical Implications and Future Directions

The evolving therapeutic landscape of UC highlights the need for evidence-driven personalization of treatment. Future research should prioritize head-to-head trials comparing oral small-molecule therapies with biologic agents to clarify relative efficacy, safety, and patient-reported outcomes [10]. The integration of biomarker-guided therapy selection, including inflammatory, genetic, and microbiome-based predictors of response, may further refine treatment decisions and reduce trial-and-error prescribing. Long-term safety surveillance remains essential, particularly for newer oral agents with systemic immunomodulatory effects. Ultimately, these advances will support a shift toward personalized medicine in UC, where therapy selection is informed by disease characteristics, patient preferences, mechanistic considerations, and long-term risk profiles rather than a fixed treatment hierarchy.

## Conclusions

This systematic review highlights an evolving shift in the therapeutic landscape of moderate-to-severe UC by demonstrating that oral small-molecule therapies are not merely adjunctive options but viable, mechanism-driven alternatives to established biologic agents. By synthesizing evidence from eight landmark publications encompassing multiple pivotal phase 3 randomized controlled trials across diverse therapeutic classes, this review underscores that treatment efficacy and safety profiles vary according to mechanism of action and treatment phase, supporting a more individualized and goal-oriented approach to therapy selection. The findings emphasize the importance of aligning treatment choice with clinical priorities such as rapid disease control, long-term remission durability, safety considerations, individualized risk stratification, and patient preference. Collectively, this work provides a clinically relevant framework for therapeutic positioning and mechanism-based treatment sequencing in UC, reinforcing the growing importance of personalized decision-making in contemporary clinical practice.
